# Enhanced sensitivity to odors due to chemosignals associated with anxiety

**DOI:** 10.1038/s42004-025-01512-3

**Published:** 2025-04-29

**Authors:** Annkatrin Wunder, Nele Bürkel, Imke Guder, José I. Zorzin, Christiane Mühle, Helene M. Loos, Jessica Freiherr

**Affiliations:** 1 Friedrich-Alexander-Universität Erlangen-Nürnberg (FAU), Department of Psychiatry and Psychotherapy, Schwabachanlage 6, 91054 Erlangen, Germany; 2 Friedrich-Alexander-Universität Erlangen-Nürnberg (FAU), Chair of Aroma and Smell Research, Department of Chemistry and Pharmacy, Henkestrasse 9, 91054 Erlangen, Germany; 3 Friedrich-Alexander-Universität Erlangen-Nürnberg (FAU), University Hospital Erlangen, Department of Conservative Dentistry and Periodontology, Glueckstrasse 11, 91054 Erlangen, Germany; 4 Fraunhofer Institute for Process Engineering and Packaging (IVV), Giggenhauser Str. 35, 85354 Freising, Germany; 5 Fraunhofer Institute for Process Engineering and Packaging (IVV), Sensory Analytics and Technologies, Giggenhauser Str. 35, 85354 Freising, Germany

**Keywords:** Mass spectrometry, Small molecules, Chemical ecology

## Abstract

Chemocommunication is important in human nonverbal communication. Behavioral effects of anxiety chemosignals on recipients are established but it is unknown whether odor sensitivity can be modulated by such stimuli. We tested recipients’ sensitivity to phenylethyl alcohol (PEA) and n-butanol after exposure to anxiety chemosignals, neutral chemosignals and blank condition. As fourth condition, a horror movie was shown, to compare the effects of visual stimuli and chemosignals on odor sensitivity. Odor sensitivity for PEA was significantly increased by anxiety chemosignals compared to blank condition. No change in odor sensitivity was evident for n-butanol. We also investigated the composition of the applied chemosensory stimuli using untargeted one-dimensional gas chromatography-mass spectrometry (GC-MS) and -olfactometry (GC-O). Considering GC-MS data, several compounds were found with significantly higher normalized peak areas in anxiety sweat samples compared to the neutral samples. GC-O analysis indicated that the carboxylic acids dodecanoic acid and 3-hydroxy-3-methylhexanoic acid were potential main contributors to the odor of anxiety sweat samples, whereby tetradecanoic acid and patchouli alcohol were those of the odor of neutral sweat samples. This study provides evidence that anxiety chemosignals can improve odor sensitivity and constitutes a further step toward elucidating the composition of sweat samples used in chemical communication research.

## Abstract (web summary)

Anxiety chemosignals increase cognitive performance and enhance sensory acquisition behavior, however, their impact on odor sensitivity is unknown. Here, the authors utilized a combination of behavioral experiments and chemical analyses to explore odor sensitivity to phenylethyl alcohol (PEA) and n-butanol after exposure to both anxiety and neutral chemosignals, revealing increased odor sensitivity for PEA.

## Introduction

The composition of body odors varies between individuals, and even within individuals it differs depending on the individual’s condition. Through this variation, body odors convey trait- and state-dependent information, related to genetics, age, sex, hygienic and nutrition habits as well as health status, reproductive state, or experienced emotions^1^, and thereby play a decisive role in non-verbal communication between individuals. Chemical cues and signals (in the following: chemosignals) related to different emotional conditions influence the behavior of recipients^2,3^. For example, chemosignals in axillary odor samples obtained from fearful individuals increase cognitive performance of recipients. Participants can solve tasks more accurately while smelling fear sweat compared to either neutral sweat or a control odor^4^. Anxiety chemosignals also increase the amplitude of the startle reflex, pointing to pre-attentive priming of defensive behavior through smelling anxiety sweat^5^. Further, anxiety signals activate areas of the brain that are associated with the regulation of emphatic feelings and the emotional control system^6^. Taken together, those studies suggest that fear and anxiety chemosignals enhance sensory acquisition behavior leading to a faster and more accurate reaction in a dangerous situation^7^. Therefore, we hypothesized that fear and anxiety chemosignals also modulate perception of environmental odors, manifested in a higher olfactory sensitivity. Accordingly, with the present study, we aimed to explore if the perception of anxiety chemosignals leads to an increase of sensitivity for environmental odors. We further aimed to compare the effects of chemosignals with those of a complex visual stimuli. An influence of visual stimuli with emotional content on odor perception has been demonstrated earlier: the emotional state of men modulated the perceived intensity of odorants^8^.

Odor sensitivity of the participants was determined for the environmental odors PEA and n-butanol. Phenylethyl alcohol (PEA) and n-butanol were selected because they are common odors with documented odor threshold distributions^9^. Both odors are used as standards in olfactory research^10,11^. Further, established tests for odor threshold determination were already available for those odors^12^.

Distinguishing between fear and anxiety is often difficult, especially in the context of chemosignals. According to the American Psychiatric Association (APA) fear is a strong biological response to immediate threat, while anxiety is an emotional overreaction to a situation that is only subjectively seen as threatening whereby there is no danger to life and limb. Notwithstanding the differences between these two emotional states, a recent fEMG study showed a similar activation pattern of the medial frontalis muscle during exposure to fear and anxiety chemosignals. In both cases, the recipients are prompted to increased vigilance^13^. Various methods, most commonly oral exams where the test anxiety of negative consequences or failure is utilized^14^, are described in the literature to obtain anxiety chemosignals. In our study we used chemosignals obtained during a dental treatment of participants being afraid of the dentist.

The term dental anxiety has been used to describe different states, ranging from a general feeling of fear and apprehension^15^ to an extreme or disproportionate fear^16^ and in extreme cases even to dental phobia^17^. Concerning the difference between dental fear and anxiety, they are often used interchangeably or combined as dental fear and anxiety (DFA)^18,19^. In this study, the chemosignals sampled are referred to as anxiety chemosignals, as we assume that the state of anxiety predominated. For the neutral chemosignal condition, body odors collected during the night were used as samples collected at night are considered to be less variable, participants’ emotional and physical states fluctuate less, and sampling conditions can be better controlled^20^.

The chemosensory stimuli used in this study were further characterized in terms of their volatile organic compound (VOC) and odorant composition. Whereas the general biochemical principles of axillary odor formation have been elucidated^21,22^, little is known about the chemical nature of chemosignals of fear, anxiety, or related states. Lately, Tsukuda et al.^23^ examined the volatile patterns emitted from the axilla and hand of 30 women who participated in a Trier Social Stress Test (TSST). Six potential stress markers in the emissions from the axilla, namely 1,2-ethanediol, acetophenone, heptadecane, hexanedioic acid dimethyl ester, benzyl alcohol and benzothiazole were reported. Vautz et al.^24^ indicated that pentadecane, 4,6-dimethyl-dodecane, dodecanal, 1-dodecanol, hexadecane and tetradecanal were present in higher concentrations in axillary sweat samples collected during the TSST condition compared to a control exercise condition. Further, Smeets et al.^25^ identified several linear aldehydes and ketones in relatively higher concentrations in fear sweat samples. These exemplary results illustrate that current evidence on qualitative and quantitative correlates of an experienced emotion in sweat composition is contradictory and fragmentary.

Our hypothesis was that smelling anxiety chemosignals can, like visual anxiety induction, lead to an increase in odor sensitivity. To test the hypothesis, and to achieve insights into the sensory characteristics and chemical composition of the applied chemosensory stimuli, we combined behavioral experiments, odor profile analysis by a trained panel and instrumental analysis of odorants (gas chromatography-olfactometry) and volatiles (gas chromatography-mass spectrometry).

## Results

### Donation of chemosignals

A total of 24 axillary chemosignal samples from anxious (A) and emotionally neutral states (N) were collected from the left and right underarms of 12 females with dental anxiety (mean age = 41.0 years, SD = 12.5 years). All donors showed increased dental anxiety, evaluated via the Hierarchical Anxiety Questionnaire HAF^26^ for dental anxiety and low, moderate and high values in the STAI for trait anxiety. Pooled samples were used to investigate their effects on odor sensitivity in a receiver cohort (see Methods for details).

### Behavioral study: influence of chemosignals on odor thresholds

Odor thresholds of PEA and n-butanol were determined in 18 men and 18 women after exposure to anxiety chemosignals (A), neutral chemosignals (N), a chemosensory control condition (blank: B), and a horror movie (H), on four different days (see Methods for more details).

#### Odor sensitivity for PEA but not n-butanol is influenced by chemosensory context

**Fig. 1:**
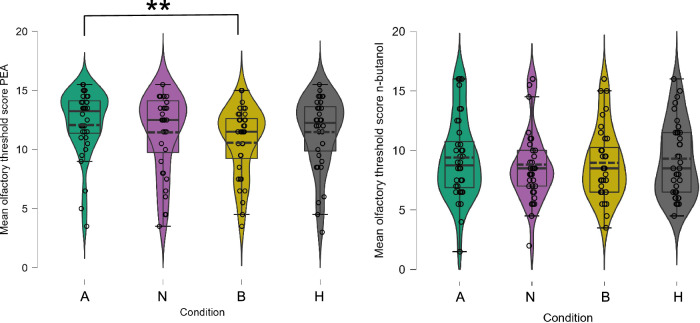
Mean olfactory threshold scores for phenylethyl alcohol (PEA) and n-butanol. The olfactory threshold scores are displayed separatly for test conditions anxiety chemosignals (A), neutral chemosignals (N), blank (B) and horror movie (H). Error bars represent 95% confidence intervals; - - - = mean, — =median; *n* = 36 (18 female, 18 male). Bonferroni correction was applied in the analysis.

Tests of within-subject effects were applied using an rmANOVA with the fixed factor *condition* (A, N, B, H) separately for both olfactory threshold scores. Within the rmANOVA for PEA, a significant shift in odor threshold (F (3, 105) = 3.681, *p* = 0.014, η^2^ = 0.095) was detected. Post-hoc tests revealed a significant difference in odor thresholds between the anxiety chemosignal condition (mean_OTS_ = 12.3) and blank condition (mean_OTS_ = 10.7) (*p* = 0.008, Cohen´s d = 0.500; Fig. 1) with the anxiety chemosignals leading to a higher odor sensitivity. All other post-hoc comparisons were not significant. In the rmANOVA for n-butanol, no significant differences between conditions became evident (F (3, 105) = 0.325, *p* = 0.807).

#### Intensity and pleasantness of PEA and n-butanol are independent of chemosensory context

It was tested whether the chemosignals can influence the perception of intensity or pleasantness rating of the environmental odors. Overall, the odors of PEA and n-butanol (most intense Sniffin’ Stick in each case) were rated significantly differently from each other concerning intensity (F (1,35) = 8.269, *p* = 0.007, η^2^ = 0.063) and pleasantness (F (1,35) = 75.149, *p* < 0.001, η^2^ = 0.517). The odor of PEA was perceived less intense and more pleasant in comparison to n-butanol. For PEA, neither intensity (F (3,105) = 1.726, *p* = 0.166) nor pleasantness (F (3,105) = 1.481, *p* = 0.224) ratings differed significantly between conditions. For n-butanol, there were no significant differences in intensity (F (3,105) = 0.625, *p* = 0.601) between conditions, but in pleasantness (F (3,105) = 2.839, *p* = 0.042, η^2^ = 0.075). Posthoc tests, however, did not reach significance (Table 1).

**Table 1:** Intensity and pleasantness ratings of PEA and n-butanol

	Odorant	Mean_Anxiety_ [SD]	Mean_Neutral_ [SD]	Mean_Blank_ [SD]	Mean_Horrormovie_ [SD]
Intensity	PEA	63.1 [20.7]	61.2 [17.4]	61.8 [18.2]	68.1 [17.1]
	n-Butanol	73.1 [13.9]	69.3 [18.1]	69.8 [16.6]	71.2 [18.4]
Pleasantness	PEA	74.0 [21.9]	72.4 [23.3]	76.9 [20.0]	71.5 [22.3]
	n-Butanol	44.1 [20.2]	43.1 [20.6]	39.2 [17.3]	35.5 [19.4]

The ratings were provided on VAS (0–100) for the conditions anxiety chemosignals, neutral chemosignals, blank and horror movie.

*SD* standard deviation.

### Chemical study: odor profiles and composition in volatiles and odorants

#### Anxiety and neutral chemosignals do not significantly differ in their odor profile

**Fig. 2:**
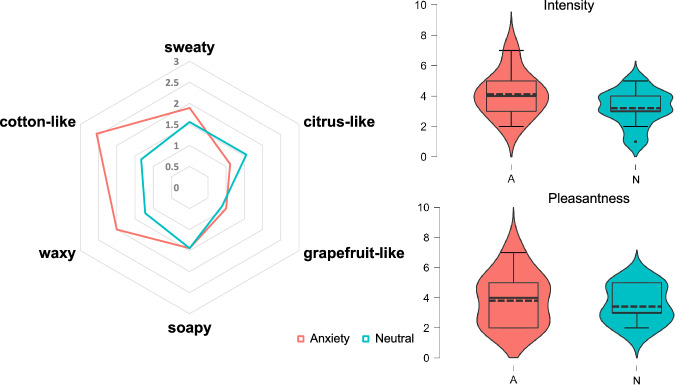
Odor profiles (left) and ratings of intensity and pleasantness (right) of the chemosignal samples. Anxiety chemosignals are depicted  in red and neutral chemosignals in blue. The data are displayed as mean numerical values of the orthonasal sensory evaluation (*n* = 9 panelists). The scale ranged from 0 (no perception) to 10 (very strong perception) for intensity ratings, and from 0 (dislike) to 10 (like) for pleasantness ratings. Error bars represent 95% confidence intervals; - - - = mean, — =median.

A trained panel determined odor attributes to establish and compare the overall odor profile of anxiety and neutral chemosignals. In the orthonasal sensory evaluation, six odor qualities were selected for the description of the perceived smells (Fig. 2 and Supplementary Table S1): sweaty, citrus-like, grapefruit-like, soapy, waxy and cotton-like. Slight variations between the samples were observed for the waxy and citrus-like and cotton-like impressions while the perceived intensities of all other attributes were comparable. The overall odor intensity was rated on average 4.1 (SD = 1.5) for anxiety and 3.2 (SD = 1.2) for neutral chemosignals (0: no perception, 10: very strong perception), with pleasantness values of 3.9 (SD = 1.8) and 3.6 (SD = 1.2; 0: dislike, 10: like). An rmANOVA with the factors condition and descriptor showed that the odor profiles were not significantly different between conditions (F (1,8) = 2.215, *p* = 0.175). A dependent t-test also showed no significant differences in the total intensity (*p* = 0.121) nor pleasantness rating (*p* = 0.347).

#### Odorants in anxiety and neutral chemosignals were elucidated via GC-O analysis

Solvent extracts obtained from anxiety and neutral chemosignals and blank samples were analyzed using GC-O. To screen for potent odorants in the samples, one pooled sample was analyzed per sample type (anxiety, neutral, blank). In total, 51 compounds were olfactorily detected in the anxiety sample, 44 in the neutral sample and 34 in the blank sample. The five compounds with the highest odor dilution (OD) factors of either the anxiety or neutral sample (Table 2) were identified as decanoic acid (fatty, cardboard-like, soapy), dodecanoic acid (waxy, soapy), tetradecanoic acid (green, green bell pepper-like), 3-hydroxy-3-methylhexanoic acid (mouldy, sweaty) and patchouli alcohol (earthy). The latter two were tentatively identified via odor and retention indices on two columns because concentration was too low to obtain a mass spectrum (see Table 2). Tetradecanoic acid and patchouli alcohol were present with higher OD factors in the neutral sample and dodecanoic acid and 3-hydroxy-3-methylhexanoic acid had higher OD factors in the anxiety sample (see Table 2). Decanoic acid was perceivable in the highest dilution in both conditions.

**Table 2:** Potent odorants in chemosignal samples, detected by means of gas chromatography-olfactometry and -mass spectrometry

Proposed compound	odor attribute	RI FFAP	RI DB-5	FD _anxiety_	FD _control_	FD _blank_	Identification method
Patchouli alcohol	Earthy	2136	1681	256	1024	4	O, RI, Std
Decanoic acid	Coriander-like, plastic-like, soapy	2241	1368	1024	1024	16	O, RI, MS, Std
3-Hydroxy-3-methyl hexanoic acid	Mouldy, sweaty	2330	1167	1024	64		O, RI, Std
Dodecanoic acid	Waxy, soapy	2455	1565	1024	4		O, RI, MS, Std
Tetradecanoic acid	Green, green bell pepper-like	2664	1756	64	1024		O, RI, MS, Std

Odor description as provided by trained panelists. FD-factors on DB-FFAP column. Compounds were identified by: *O* Odor quality, *RI* retention indices on DB-5 and DB-FFAP columns, *MS* mass spectrum, *Std* comparison with reference, *n.d.* not detectable.

#### GC-MS data provides hints on different composition of anxiety and neutral chemosignals

**Fig. 3:**
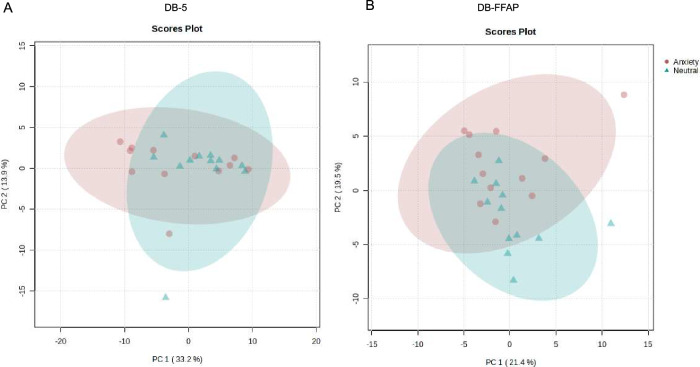
Results of the principal component analysis of the chemosignal samples. Scores Plot PC 1 vs. PC 2 for pareto scaled GC-MS data of anxiety (red circles) vs. neutral (blue triangles) chemosignals on DB-5 (**A**) and DB-FFAP (**B**) column; *n* = 12 participants (127 features for DB-5 and 58 features for DB-FFAP analysis).

Anxiety and neutral chemosignals (*n* = 12 per condition) were solvent extracted and analyzed using GC-MS equipped with two different columns (DB-5 and DB-FFAP; see Methods for details). To visualize the relation between the composition of anxiety and neutral samples, principal component analysis (PCA) was performed. The PCA’s scores plot revealed on both columns a partial overlap of anxiety and neutral samples (see Fig. 3). The volcano plot revealed features with higher levels in anxiety samples as well as neutral samples (see Supplementary Fig. S1). The features were tentatively identified using the NIST MS library (Table 3a, b).

**Table 3:** NIST matches and normalized mean area of significant (*p*< 0.01) features in anxiety and neutral chemosignals based on GC-MS analysis with DB-5 column (a) and DB-FFAP column (b)

Feature	NIST match	MF	RM	Probability [%]	Mean area _Anxiety_ [SD]	Mean area _Neutral_ [SD]
**a DB-5 column**						
9	4-Methyl-octane	885	923	57.07	1.18 [2.63]	0.82 [2.17]
11	No match				0.31 [0.42]	0.20 [0.38]
131	Octadecyl (Z)-hexadec-9-enoate	846	855	23.46	7.87 [8.16]	15.29 [8.25]
**b DB-FFAP column**						
23	2-(2-Ethoxyethoxy)-ethanol	830	874	84.49	3.15 [1.47]	1.40 [1.06]
24	1,2-Ethanediol	858	925	93.36	1.87 [1.06]	0.81 [0.61]
26	2-Hydroxypropyl 2-methylprop-2-enoate	814	866	78.16	0.39 [0.32]	0.07 [0.05]
27	No match				0.88 [0.82]	0.31 [0.32]
29	No match				4.11 [2.54]	1.75 [1.30]
31	Benzyl alcohol	811	851	30.67	1.49 [0.65]	0.70 [0.54]
37	No match				0.96 [1.17]	0.38 [0.47]

Mean areas normalized by sum. Individual feature areas can be viewed in the supplementary data sheet S1.

*MF* Match Factor, *RMF* reversed Match Factor, *SD* standard deviation.

## Discussion

Our research shows that anxiety chemosignals increase odor sensitivity of the recipients, as sensitivity for PEA was significantly higher in the anxiety condition compared to the non-body odor blank condition. However, odor sensitivity was only increased for PEA and not for n-butanol. Also, no significant difference in odor sensitivity was found when comparing the anxiety with the neutral chemosignal condition.

In humans, it is suggested that in fearful situations the release of adrenalin via the sympathetic-adrenal medullary system induces the production of anxiety chemosignals, triggering the fight-or-flight response in the emitter and receiver^27^. The chemosignals modulate thereby the activation of the amygdala and hypothalamus in the receivers, which are closely linked to behavioral and endocrine responses^28^. In addition, a growing number of studies uncovered how exposure to chemosensory fear signals can increase anxiety in recipients and affect their processing of social information, such as facial expressions and impression formation^29,30^. De Groot et al.^31^ discovered that the change in facial expression due to fear chemosignals is associated with an increased sniff magnitude. Similar effects of sensory sharpening have also been observed with other senses as studies have shown that wide-open eyes, which typically signal fear, increase our field of vision, making it easier to recognize and localize threats^32^. An anxiety-induced dilution of nostrils has not been experimentally demonstrated yet, but is conceivable based on the results of the visual sense. Flared nostrils would allow more odorous molecules to enter the nasal cavities. If the emotion of fear or anxiety is to some degree communicated to the recipients of the chemosignals, it can be assumed that these signals foster the emergence of mechanisms that help to cope in threatful situations. An improved odor perception, as we reported here, is in line with this notion, as it indicates an enhanced sensory acquisition behavior, which can be crucial for recognizing threats and initiating a fight-or-flight response. This, however, would not explain why a change in odor sensitivity could be observed for PEA only.

In previous research, a positive correlation between odor sensitivity and salivary cortisol levels (increased by stressful or anxiety situations) has been demonstrated^33^. This is consistent with studies showing that women with elevated cortisol levels are better in perceiving and recognizing odors^34,35^. This indicates that experienced stress or social anxiety leads to an increased odor sensitivity. The exact mechanisms are still unclear; however, it is postulated that the increased olfactory abilities could be explained by an increased activation of the amygdala during stress^36^. Whether anxiety chemosignals can increase the recipients’ salivary cortisol levels has not yet been investigated, but in general, axillary stimuli have already been shown to modulate cortisol levels^37,38^. Therefore, it can be hypothesized that an increase in cortisol levels induced by anxiety chemosignals might mediate the increased odor sensitivity that we observed for PEA. Odor sensitivity was not increased in our study in the horror film condition even if the participants showed increased anxiety measured by STAI and PANAS (see SI Methods). From this it can be concluded that the response to anxiety transmitted by chemosignals differs significantly from anxiety induced by the horror movie stimulation. The reason for this may be the different processing of the two stimuli. The stimulation with the horror movie is a complex stimulation in which, in addition to visual stimulation, sounds and movements are used to intensify the experienced anxiety. In contrast, the stimulation with the anxiety chemosignals is mainly an odor stimulation, even if additional information can also be transmitted through the chemosignals. For other emotions such as aggression, no improvement, but rather a reduction in odor perception was demonstrated in other studies^33^. As anxiety is a multifaceted emotion that often also involves other feelings such as fear or stress, it is conceivable that the observed differences are also based on a different mix of emotions. Especially as the perceived emotions watching horror movies are complex and range from fear to joy^39^.

Also, for clinical forms of anxiety in patient groups, it is known that olfactory performance decreases in a fearful state. Takahashi et al.^40^ showed that high STAI ratings were significantly correlated with reduced olfactory functioning, especially for the recognition of PEA. In another study young adults with generalized anxiety disorder showed deficits in odor threshold, discrimination, and identification in comparison to healthy controls^41^.

Odor sensitivity was increased for PEA but not for n-butanol. This could have diverse reasons. First, PEA was perceived as more pleasant than n-butanol. Sniffing behavior is modulated by perceived pleasantness^42^; accordingly, the effect of more dilated nostrils due to anxiety chemosignals may be abolished in the case of n-butanol. Second, the variance of the observed odor sensitivity is greater for n-butanol, making it more difficult to observe overall effects. Third, different mechanisms might occur on a peripheral level, like odorant-odorant competition for enzymatic binding sites leading to increased nasal availability and consequently sensitivity^43^, or adaptation leading to decreased sensitivity. Short-chain aliphatic alcohols, acids and aldehydes are known constituents of body odor^25,44,45^, and might lead to a certain adaptation to n-butanol and thus neutralize the effect of the anxiety chemosignals. Even though it is discussed that chemosignals do not bind to the regular odor receptors and have their own system of signal transmission [reviewed by Precone et al.^46^], other compounds in sweat^47^ may lead to such adaptation. To resolve this, further studies with more odorants differing in their pleasantness and chemical structures are needed. Thereby, testing threat-related odors like odors of fire, dangerous animals or rotten food would be particularly interesting in view of their potential ecological significance.

No significant difference in odor sensitivity for PEA nor for n-butanol was found when comparing the two body odor conditions (anxiety and neutral chemosignals). Visual inspection of the data nonetheless suggests that anxiety samples increase sensitivity more than the neutral samples and that the effect might be too small to be shown within our sample size. Also, the neutral samples were taken the night before the dentist appointment and it cannot be completely ruled out that the participants did not already experience any anxiety, thinking about their dental appointment on the next day. This should be seen as a limitation of this study, and future studies should consider a longer timeframe between sampling points and aim to obtain control samples from an ideal control condition (e.g. same duration of sampling). Additionally, repetition with a higher number of participants and odorants is needed to fully uncover the effects of human chemosignals on odor sensitivity. The use of chemosignals gained through a strong emotional deflection, like here from people with dental anxiety, could be decisive in such future studies. In the present study, the donors showed only moderate anxiety during the dental treatment. As donors we used females, in contrast to previous studies mainly using male body odors. This and the fact that we used a within-subject design are methodological strengths and sets our study apart from others.

Pleasantness and intensity ratings did not change due to chemosignals. This result is contrary to the study by Ferreira et al.^48^ as here neutral chemosignals were perceived as more intense following the presentation of chemosignals of fear and disgust. Hence, currently there is no evidence for an influence of anxiety chemosignals on the intensity and hedonic ratings of environmental odors (present study), but on chemosignals. In future studies, different chemosensory stimuli (e.g., differing in their pleasantness, intensity) should be considered both as priming and target stimuli.

Chemical analyses were conducted to evaluate potential differences in the composition of the chemosensory stimuli applied here. This served two purposes: to get insights into i) compounds potentially involved in chemical communication of emotional states, and ii) the nature of odorants and volatiles presented to the participants in the present study (quality control). Exploratory GC-O analysis of one pooled sample per condition indicated that the odor composition of the anxiety and the neutral sweat samples differed. Tetradecanoic acid and patchouli alcohol appeared to be more important in the neutral condition and dodecanoic acid and 3-hydroxy-3-methylhexanoic acid in the anxiety condition. 3-Hydroxy-3-methylhexanoic acid is a compound that is frequently detected in sweat and is thought to play an important role in chemo-communication^49,50^. So far, however, it has not been associated with stress sweat. Dodecanoic acid was already identified as a potential physiological stress biomarker in human sweat^51^. These compounds may thus be related to emotional sweating. Patchouli alcohol probably stems from perfume or scented product use and probably occurs in higher amounts in the neutral condition due to the longer sampling duration and different sampling environment (the homes of the participants). The longer sampling duration may also be the reason why the OD factors were higher for tetradecanoic acid. Yet, further studies are needed to confirm the here obtained semi-quantitative results via quantification of the compounds of interest. Comparing results of the GC-O analysis and odor profile analysis at first glance, these appear to be contradictory (differences in odorant composition but not in odor profiles). There are different possible explanations. In the GC-O analysis, the analytes are chromatographically separated before smelled with the nose, whereas in the odor profile, all components are smelled simultaneously. This can lead to addition or subtraction effects influencing the overall odor impression. Overall, the samples were not very intense regarding their smell and the matrix generally influences odor thresholds. Therefore, it cannot be excluded that odorants were below their odor threshold in the body odor samples, but could be perceived during GC-O.

In the analysis of individual samples using GC-MS, significant differences in the volatile profiles became evident. Particularly when measuring on the DB-FFAP column, several features occurred with significantly higher normalized peak areas in the anxiety sweat samples in comparison to the neutral sweat samples. The features 24 and 31 (DB-FFAP), tentatively identified as 1,2-ethanediol and benzyl alcohol, were already reported as stress markers in skin gases by Tsukuda et al.^23^. All other features listed in Table 3a, b have not yet been described in the literature as components of human sweat.

Cotton materials have been successfully used to sample body odors and demonstrate their effects on receivers, and this is why they were also used in this research. Nonetheless, they have several limitations, and future research should also consider alternative sampling methods, like sampling on specific sorbent materials^52^. For instance, it cannot be excluded that the hydroxyl functional groups of the cellulose retain hydrophilic compounds from the sweat^53–55^.

Taken together, we demonstrated that odor sensitivity can be increased by anxiety chemosignals. Additionally, hints towards a different chemical composition of anxiety and neutral body odor samples were obtained. This study therefore provides the basis to further evaluate the modulation of odor sensitivity by one’s own experienced emotion or by emotions experienced by others, as conveyed via chemosignals, and to further elucidate the molecular basis of chemical communication of emotions in humans.

## Methods and material

The entire study protocol was approved by the Medical Faculty Ethics Review Board of the Friedrich-Alexander-Universität Erlangen-Nürnberg (no. 22-277-B) and was conducted in accordance with the Declaration of Helsinki between November 2022 to November 2023. Written informed consent was obtained by all participants. Detailed information regarding used chemicals and sample materials as well as ancillary information regarding the study design and donor cohort can be found in the Supplementary Material and Methods. The sample size for the donation and behavioral study was each based on the number of participants in comparable studies. A statistical power analysis using effect sizes of previous research was carried out.

### Donor study: sampling of anxiety and neutral chemosignals

#### Participants

Chemosensory samples were obtained from the axillary area of 12 female participants (mean age = 41.0 years, SD = 12.5 years), who took part in the neutral (night sweat) and anxiety condition (dental treatment). All participants were healthy, non-smokers and were neither pregnant nor breastfeeding. One participant took high blood pressure medication. Detailed demographic parameters of the donor group can be viewed in Supplementary Table S2.

#### Sampling procedure

The sampling was carried out at the Dental Clinic of the Friedrich-Alexander-Universität Erlangen-Nürnberg. The chemosignals were sampled under standardized conditions by wearing a t-shirt with gazin pads and cotton pads sewn into the shirt. The anxiety chemosignals were taken during a regular dental treatment. The participants were, independent of their study participation, treated in the dental clinic of the Friedrich-Alexander-Universität Erlangen-Nürnberg in the department of tooth preservation (dental clinic 1), dental prosthetics (dental clinic 2) or oral and maxillofacial surgery. During their treatment, participants had to wear the previously distributed T-shirts. The gazin gauze pads (for chemical analysis) as well as the cotton pads (for behavioral analysis and odor profiles) were removed immediately after collection and frozen at −80 °C. Cotton pads were cut into eight pieces before freezing them. For the neutral condition, sweat samples were taken during the night before the dental treatment. For this purpose, the participants put on the T-shirt before they went to bed and took it off when they got up in the morning. The participants put the used T-shirt in the Ziplock bag provided and handed the sample over to the experimenter before the dental session. Here, the sampling materials (gazin gauze pads and cotton pads) were also removed immediately and frozen at −80 °C. Cotton pads were cut into eight pieces before freezing.

The affective dimension of anticipatory anxiety and stress was assessed via the stress hormone cortisol in saliva and via the PANAS before the dental treatment as well as before the neutral condition. Therefore, approximately 1 ml of saliva was sampled in Salivettes (Sarstedt, Germany) and analyzed via an enzyme immunoassay for cortisol in saliva (IBL, Germany; standard range: 0.15–30 ng/mL, enzyme conjugate: cortisol conjugated with HRP, substrate: TMB). Analysis of the cortisol levels was conducted at the Department of Psychiatry and Psychotherapy at the Universitätsklinikum Erlangen. Results suggest a successful deflection as measured cortisol levels were higher even if not significant for the anxiety condition (*p* = 0.130, Cohen’s d = 0.342). The results of the PANAS support this as negative affective states were higher (T = −2.11, *p* = 0.029, Mean_Anxiety_ = 23.17, Mean_Neutral_ = 20.75) before the dental treatment.

### Behavioral study: Influence of chemosignals on odor threshold

#### Participants

For the investigation of the influence of chemosignals on odor sensitivity, 36 participants (18 females, mean age = 24.1 years, SD = 2.9 years) were invited. All participants were healthy, normosmic, non-smokers and were neither pregnant nor breastfeeding, nor took any medication. Female and male participants were matched by age and there were no significant differences with respect to body mass index (BMI), Beck depression index (BDI), trait anxiety score (STAI_Trate_), relationship status and performance in the odor identification test (see Supplementary Table S3). Detailed information on the donation of the chemosenory stimuli can be found in the SI.

#### Experimental procedure

The odor threshold measurements were carried out in the Department of Psychiatry and Psychotherapy at the hospital of Friedrich-Alexander-Universität Erlangen-Nürnberg. Participants took part in four test sessions on four separate days within four weeks. The four experimental conditions were: anxiety chemosignals A, neutral chemosignals N, blank B and horror movie H (see below).

The order of the test sessions was randomized in a balanced manner. On the first day of testing, participants provided informed consent and filled out questionnaires concerning demographic and medical [Beck Depression Inventory BDI^56^, STAI_Trait_] parameters. In addition, general odor identification performance was tested using the 16 Sniffin’ Sticks identification test [Burkhart, Germany;^57^]. After the identification test, participants filled out STAI_State_ and PANAS. Then, they were either shown one part of the horror movie or presented with an odor sample (condition A, N or B). For the presentation of the chemosignal and the blank samples participants were asked to already put on the sleep mask for the subsequent odor threshold test. This was done to ensure that participants were blinded to the fact that chemosignals were presented to them. The chemosignal samples were presented to them for 3–5 min while the procedure of the following threshold test was explained. Stimuli were presented in beakers and were within a radius of 1 meter. Then participants started with the first threshold test. Throughout the threshold test participants wore sleep masks to better focus on the task. A total of two different odor threshold tests (PEA and n-butanol) [Burkhart, Germany;^12^] were performed, with the order of the tests being randomized in balanced manner but always left in the same order per participant. Following this procedure, order effect could be compensated. For the olfactory threshold score, the shortened Version SSP5 by Pössel et al.^58^, with only five instead of seven turning points was used. At the beginning of each threshold test, participants were presented with the pen with the highest concentrated to get familiar with the odor. Afterwards they were asked to rate the perceived intensity and pleasantness of the odor on a Visual Analog Scale (VAS) from 0 to 100. Between the two threshold tests, the odor sample or another part of the horror movie were presented again. This procedure was repeated after the second odor threshold test. Subsequently a questionnaire including STAI and PANAS was filled out. The study design of the behavioral study is displayed in Supplementary Fig. S2.

#### Complex visual stimuli

A horror movie condition was introduced to investigate the influence of experienced anxiety on the sensitivity for environmental odors. The horror movie had a length of approximately 10 min and consisted of scenes that were taken from standardized databases and previous studies [refs. ^59–61^; see also Supplementary Table S4]. The horror movie was divided into three parts of 3 min to match the length of the chemosignal presentation during the other conditions. It was shown on a screen directly in front of the participants and subjects wore headphones to block out background noise and focus on the film. To control for induced anxiety, participants rated their experienced anxiety during the horror movie from 1 to 10 (no anxiety at all to totally scared) and State-Trait Anxiety Inventory STAI^62^ and Positive and Negative Affect Schedule PANAS^63^ values before and after the movie were evaluated. The results suggest a successful fear induction by the horror movie (see Supplementary Methods).

#### Chemosensory stimuli

Three chemosensory conditions were applied. Anxiety and neutral chemosignals were presented in form of donor pools which were prepared by randomly pooling four cotton pad pieces (one piece each of four different donors). Care was taken to ensure that each donor was used the same number of times, and that no donor was used twice in a pool. Participants received the same pools from both conditions to minimize biasing effects of interindividual variability in sweat production. In addition to the two chemosignal conditions, unused cotton pads were used as a blank condition. Before the stimulus presentation, the participants were not supplied with any information about the stimuli.

#### Statistics

The data were analyzed using repeated-measures analysis of variance (rmANOVA) and Bonferroni corrected posthoc tests using JASP software (JASP Team, Version 0.16.3.0). Significant differences were assumed at *p* ≤ 0.05. Effect sizes are indicated for the major significant results (Cohen’s d). Outlier analysis using Box-Plot graphs was performed. The Shapiro–Wilk test was applied to assess the normality of the data. Normality was not given for all combinations, but as prior work showed that the violation of normality has only marginal effect on the false positive rate of the hypothesis test, we continued our analysis using parametric testing^64–66^. If a violation of sphericity using the Mauchly test was assumed, Greenhouse-Geisser correction was applied for data analysis. To control for possible order x conditions effects, the order of the odor threshold test was added as a between-subject factor. In a multivariate ANOVA including both odors, the chemosignals did not have an effect on odor thresholds per se, but a distinct effect on sensitivity for the odor PEA and not on sensitivity for the odor n-butanol occurred. We thus decided to run two separate rm ANOVAs for the two odors.

### Chemical study: odor profiles and composition in volatiles and odorants

#### Odor profile analysis

##### Sample preparation

Cotton pads were defrosted 30 min prior to their usage. Twelve pieces (one piece each of twelve different donors) per condition were presented in covered glass vessels coded with a random three-digit number to the panelists for orthonasal sensory evaluation.

##### Procedure

The odor profiles of the samples were determined by the trained panel (7 females, 2 males, aged 25–29 years) of the Chair of Aroma and Smell Research, Friedrich-Alexander-Universität (Erlangen, Germany). All panelists were trained in describing odors with an in-house developed flavor language for at least 6 months.

Sensory analyses were carried out during three sessions, whereby session one and two took place on the same day. In the first session, panelists were asked to orthonasally evaluate all of the samples individually, then to list their individual odor attributes. In the second session, they jointly discussed these attributes to agree on the main odor attributes. Finally, in the third session, each member was asked to score the intensities of the selected attributes and the overall intensity on a scale from 0 (no perception) to 10 (strong perception). Additionally, the valence of the samples was assessed on a scale from 0 (very unpleasant) to 10 (very pleasant). This procedure is based on DIN EN ISO 13299:2016.

#### Isolation and analysis of volatiles and odorants

##### Extraction of volatiles and odorants

The Gazin® gauze pads were stirred at room temperature for 30 min with 200 ml DCM to extract the volatile compounds (see SI Materials for details on supplier). The internal standard methyl octanoate was added at a concentration of 51 µg/ml. For each participant, gauze pads of the left and right axilla were extracted together and for comparison, two unused Gazin® gauze pads were processed using the same work-up technique. After removal of the pads the extract was dried over anhydrous sodium sulfate and finally concentrated to a total volume of 100 μl at 50 °C by means of rotary evaporator and micro-distillation^67^. The distillate was then stored at −80 °C for a maximum of 7 days until further analysis with GC-MS and GC-O.

For the GC-O analysis, odor extract dilution analysis (OEDA) was applied. Here the aroma extracts are stepwise diluted and each dilution is analyzed. The odor dilution factor (OD factor) of an odorant corresponds to the maximum dilution at which this odorant can still be perceived. The OD factors can be used as a rough estimate of the significance of an odorant for the overall odor of the investigated sample^68,69^ (see SI Methods).

For the GC-MS analysis, the distillates were injected in cold-on-column mode and gas chromatographic separations were performed with the capillary columns DB-FFAP and DB-5. Methodological details can be found in the SI. Peak definition and peak area integration out of the raw GC-MS data was performed based on the deconvoluted mass spectra and the NIST MS library using the PARADISe software (version 6.0.1)^70^ for each data set (FFAP and DB-5). Subsequently the resulting peak table was processed by removing features tentatively identified as silicate derivate via commercial spectral database NIST MS Search 2.0, and 5% of the features were filtered based on the interquartile range. On the DB-5 column peak areas of 148 components plus the internal standard were determined. After data cleaning 127 features remained. On the DB-FFAP column peak areas of 63 components plus the internal standard were determined. Data cleaning was performed, leading to 55 features. The data was normalized by sum and square root transformed. Additionally, Pareto scaling was applied. Principal Component Analysis (PCA) using MetaboAnalyst (version 6.0) was performed. Additionally, to identify significant area changes in features between conditions, volcano plots were plotted.

### Reporting summary

Further information on research design is available in the Nature Portfolio Reporting Summary linked to this article.

## Supplementary information

Supplementary Information

Description of Additional Supplementary Files

Supplementary Data 1

Reporting Summary

## Data Availability

The data of the behavioral study and the donor group is shared on the open science framework under the name of the corresponding author A.W. (DOI 10.17605/OSF.IO/EF2S8). Individual feature areas of the normalized GC-MS analysis can be viewed in the supplementary data sheet S1. Chromatographic data will be provided on request by author H.L.
