# Supplementary material for: Enhanced sensitivity to odors due to chemosignals associated with anxiety
Source: Commun Chem. 2025 Apr 29;8:129. doi: 10.1038/s42004-025-01512-3 (PMC12041518; doi:10.1038/s42004-025-01512-3)
Supplement: Supplementary file 1 — Supplementary Information [file 42004_2025_1512_MOESM1_ESM.pdf]

Supplementary Information for

Enhanced sensitivity to odors due to chemosignals associated with anxiety

Annkatrin Wunder[a,b*,] Nele Bürkel[a], Imke Guder[a], José I. Zorzin[c], Christiane Mühle[a], Helene M. Loos[b,d†], Jessica Freiherr[a,d†*]
† = These authors share last authorship.

*corresponding authors: Annkatrin Wunder und Jessica Freiherr
Email: annkatrin.wunder@fau.de; jessica.freiherr@fau.de

This PDF file includes:

Supplementary Material
Supplementary Materials
Supplementary Figure S1 and S2
Supplementary Tables S1 to S4
Supplementary References

Supplementary Material

Chemicals

Methanol, dichloromethane (DCM) and anhydrous sodium sulfate were purchased from VWR Chemicals (Darmstadt, Germany). DCM was freshly distilled before usage. The internal standard methyloctanoate (purity: 99%) was bought from Aldrich (Steinheim, Germany). The following reference compounds were used: decanoic acid (Aldrich, Steinheim, Germany, 98 % purity), dodecanoic acid (Aldrich, Steinheim, Germany, 98 % purity), tetradecanoic acid (Fluka, Steinheim, Germany, 98 % purity), 3-hydroxy-3-methylhexanoic acid (Sigma, Steinheim, Germany, 95% purity) and patchouli alcohol (BIOZOL, Eching, Germany, purity unknown). For the determination of retention indices, a diluted homologous series of alkanes ranging from hexane to tetratriacontane (Fluka and Sigma-Aldrich, Steinheim, Germany) was used.

Sample material

T-shirts (fruit of the loom, USA) were prewashed with ultra-sensitive laundry detergent powder (dm Drogeriemarkt, Germany). Then cotton pads (dm Drogeriemarkt, Germany) and gazin gauze pads (5 x 5 cm, 8 ply, 17 threads, Lohmann & Rauscher International GmbH & Co. KG, Germany) were sewn into the t-shirts. Gazin gauze pads were pretreated according to Alves Soares et al. [1]. Briefly, the pads were repetitively extracted with methanol. The pads were then dried at room temperature and stored in amber glass bottles until sewn into the t-shirts.

Supplementary Methods

Donation of chemosignals

Regulations regarding personal hygiene and nutrition

To ensure the integrity of the chemosignals and to prevent samples from being contaminated by environmental odors, participants were instructed to refrain from eating garlic, onions or hot chili peppers, and from using perfumed deodorants, scented creams, makeup and aftershave, or perfumes for 48 hours before the first sampling time. A perfume-free body care set consisting of a shampoo and a shower gel from Balea Med - Ultra Sensitive (dm Drogeriemarkt, Germany) was provided instead. The participants also abstained from physical activity, meals, alcoholic beverages, and non-alcoholic beverages for at least half an hour before the testing session.

Behavioral study

Evaluation horror movie – descriptive data

On average, participants showed a medium level of anxiety after watching the horror movie [mean 5.6, SD 2.4, range 1-9 on the scale from 1 – 10 (no anxiety at all to totally scared)]. A rmANOVA also revealed a significantly increased $STAI_{State}$-value (F (2.2, 77.2) = 23.852, $p < 0.001$, $\eta^2 = 0.405$). Posthoc tests confirmed this significant increase in the horror film condition compared to all other conditions (H vs. A: $p< 0.001$, Cohen´s d = -1.522; H vs. B: $p< 0.001$, Cohen´s d = -1.242; H vs. N: $p< 0.001$, Cohen´s d = -1.500). The rmANOVA for PANAS values also uncovered significant changes in negative emotions (F (3,105) = 15.054, $p < 0.001$, $\eta^2 = 0.301$). Posthoc tests revealed significant increased negative emotions during the horror movie condition compared to all other conditions (H vs. A: $p< 0.001$, Cohen´s d = -1.183; H vs. B: $p< 0.001$, Cohen´s d = -1.074; H vs. N: $p< 0.001$, Cohen´s d = -1.246). Performing correlation analysis, a weak negative correlation between anxiety after watching the horror movie and odor threshold of n-butanol (Pearson's r = -0.363, p = 0.029) and no correlation between anxiety after watching the horror movie and odor threshold of PEA (Pearson's r = 0.01, p = 0.955) was detected. Additionally, 30 participants self-reported an increased heart rate, 10 an increased sweat production and 16 an increased breathing frequency during the horror movie. Further, two participants reported a change in their body odor.

The subjects were asked specifically whether they had noticed any change in their heart rate, sweat production or breathing frequency.

Chemical study

Gas chromatography-mass spectrometry, data analysis, and identification of volatiles

Gas chromatographic separations were performed using an Agilent 7890 A GC (Agilent, Santa Clara, CA, United States) equipped with the following capillary columns: DB-FFAP or DB-5 (30 m × 0.25 mm, film thickness of 0.25 µm, J&W Scientific, Agilent Technology, Santa Clara, CA, United States). Helium was used as a carrier gas at a flow rate of 1.0 ml/min in the constant flow mode. The distillates were injected in cold-on-column mode at 40°C using a multipurpose autosampler MPS2 (Gerstel GmbH & Co.KG, Mülheim an der Ruhr, Germany), with an injection volume of 1 µl. The following temperature program was used: (i) 2 min at 35°C, heating up to 240°C with a ramp of 8°C/min, holding 240°C for 10 min for the FFAP-column and (ii) 2 min at 35°C, heating up to 300°C with a ramp of 8°C/min, holding 300°C for 10 min for the DB-5 column. Mass spectrometric data was recorded in scan mode (40–400 m/z) with an ionization energy of 70 eV using an Agilent 5975C MSD (quadrupole; Agilent Technology, Santa Clara, CA, United States). The retention index (RI-value) for each compound was calculated as described by Kováts using C6-C34 n-alkanes [2].

Odor extract dilution analysis (OEDA) and identification of odorants

OEDA was performed using a TRACE GC (Thermo Fisher Scientific Inc., MA, United States) equipped with a DB-FFAP fused silica capillary column (30 m × 0.32 mm i.d., 0.25 µm film thickness). The injection volume was 2 µL and rate of the carrier gas helium was 1.9 ml/min. the After 2 min, the temperature was raised at 8 °C/min to 240 °C (DB-FFAP), and held for 10 min. At the end of the column, the effluent was split 1:1 by a Y-splitter. Two deactivated fused silica capillaries of the same length (30 cm × 0.32 mm i.d.) led either to an FID (270 °C) or to an Odor Detection Port (ODP, 250 °C). All extracted samples were pooled per condition (anxiety, neutral) and comparatively evaluated. The samples were pooled in view of the low odor intensity and high inter-individual variety. The odor dilution factors (OD) of the odorants were determined according to Grosch (2001). Therefore, the original extract (OD = 1) of the sample was diluted stepwise (1:3; v/v) using dichloromethane, and 2 µL of each dilution were applied for GC-O analysis. Each odorant was recorded with the OD factor representing the last dilution in which the substance was still detectable. Based on the obtained OD factor, the most potent odorants, possessing the highest ODs in either the neutral or anxiety condition, were identified. Identification criteria comprised the following analytical parameters: retention indices on two columns with different polarity (DB-FFAP and DB-5), and the respective mass spectra obtained in EI mode. Mass spectrometric data obtained for the target compounds were compared with those of a commercial spectral database (NIST MS Search 2.0), and finally confirmed by the respective chemical reference standards in each case. Odor evaluation parameters (odor character and intensity) were also considered when comparing the target odorants with the respective reference substances by mass spectrometry, RI and odor impression comparison with reference compound.

Supplementary Figures

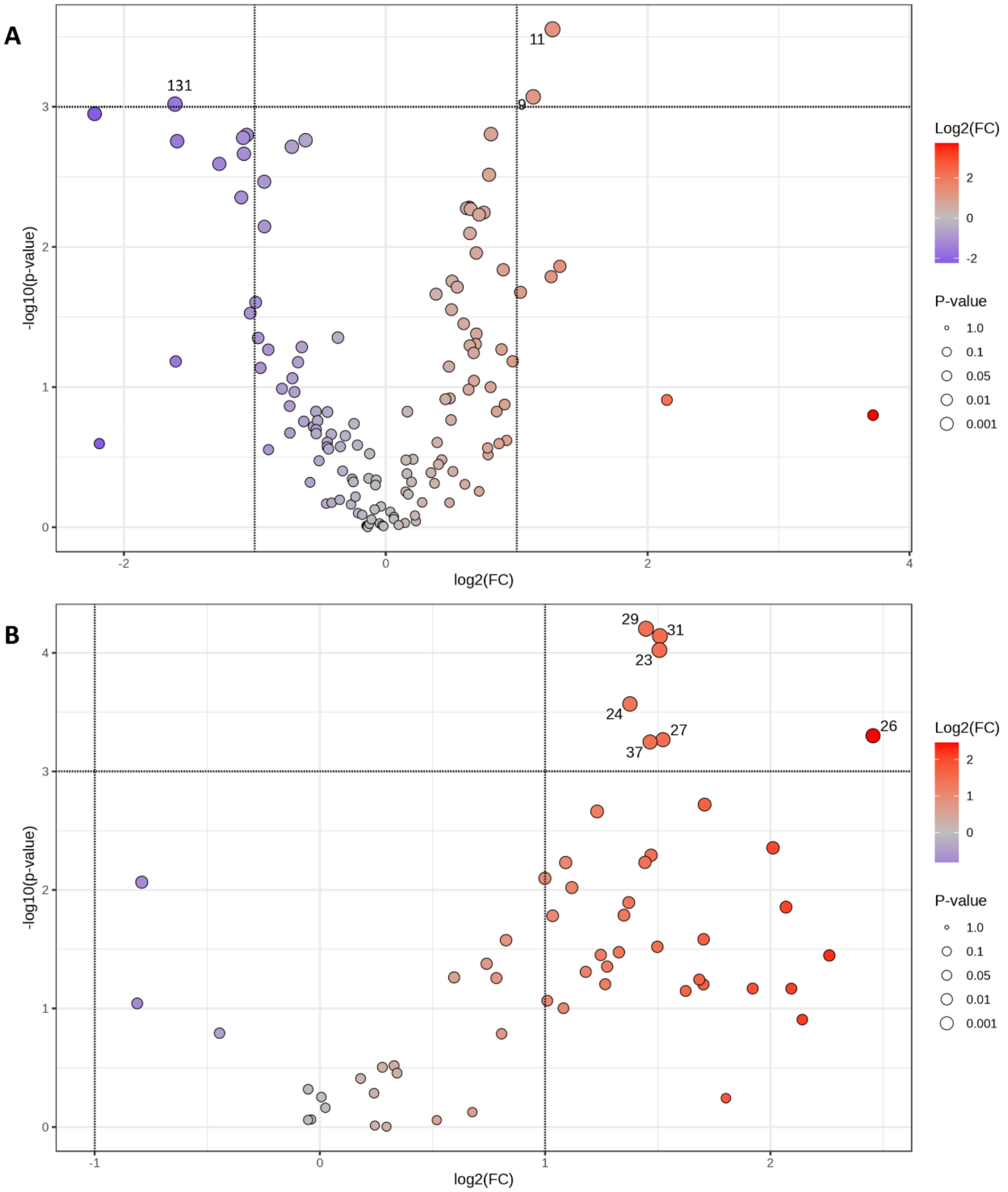

Supplementary Figure S1 Volcano plot with significant labeled features highlighted in blue (higher amount in neutral sample) on DB-5 (A) and DB-FFAP (B) column. P-value threshold$_{raw}$ = 0.001 and fold change (FC) threshold = 2.0

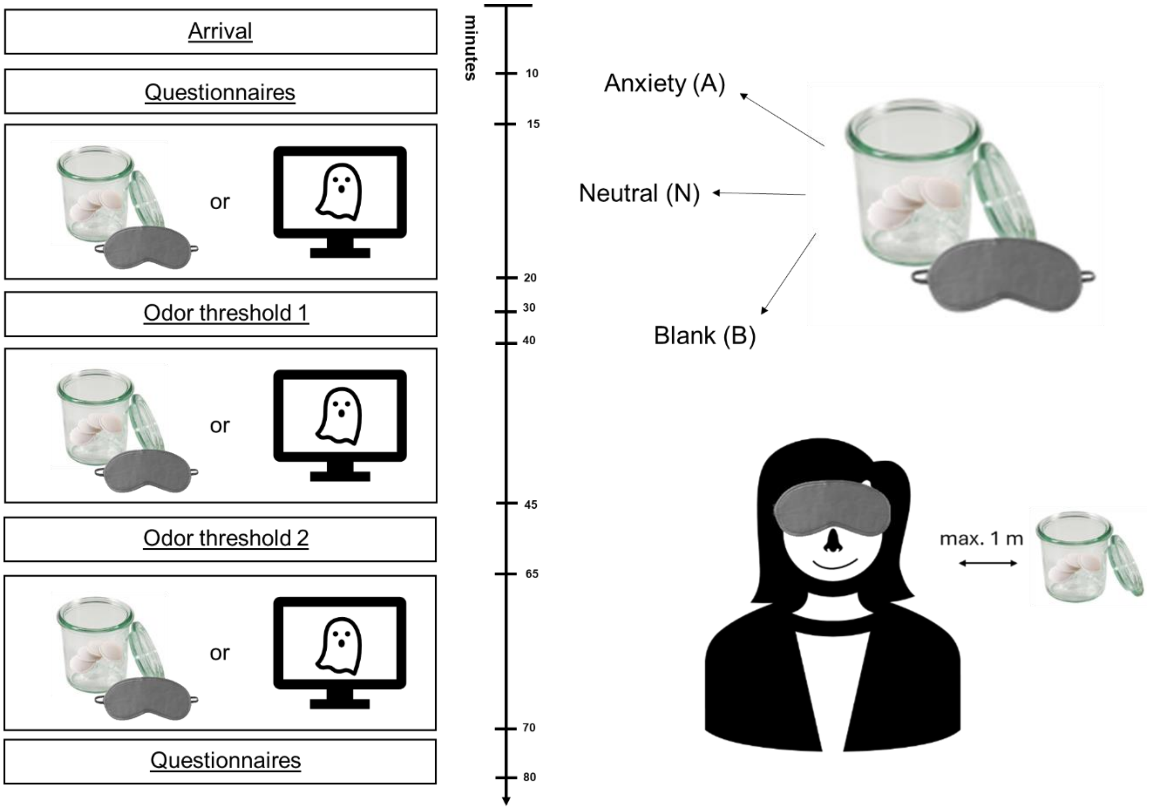

Supplementary Figure S2 Display of the study design of the behavioral study. The presentation of the body odor samples (A, N and B) took place while the participants were blindfolded, within a maximum radius of 1 meter around the participant. There were four test days, whereby on one test day only one odor condition (A, N and B) or the horror film (H) was presented.

Supplementary Tables

Supplementary Table S1. Intensity of the descriptors for the anxiety and neutral chemosignal samples.

	Mean Anxiety [SD]	Mean Neutral [SD]
Sweaty	1.9 [1.4]	1.6 [1.2]
Citrus-like	1.1 [0.8]	1.6 [1.6]
Grapefruit-like	1.0 [1.6]	0.9 [0.9]
Soapy	1.4 [1.1]	1.4 [1.3]
Waxy	2.0 [1.4]	1.2 [1.0]
Cotton-like	2.6 [1.2]	1.3 [0.9]

SD = Standard deviation.

Supplementary Table S2. Demographic parameters of donor group.

	Mean [SD]	Range
Age	41.0 [12.5]	23 - 54
BMI	24.5 [7.6]	15.4 - 41.8
$STAI_{Trait}$	37.5 [12.9]	21 - 59
HAF	36.7 [7.1]	17 - 45

SD = Standard deviation, df = 12.

Supplementary Table S3. Demographic parameters of receivers.

	$Mean_{male}$ [SD]	$Mean_{female}$ [SD]	t	p value
Age	24.7 [2.8]	23.6 [3.0]	1.161	0.254
BMI	22.9 [2.2]	21.7 [1.9]	1.682	0.102
BDI	3.6 [3.8]	4.7 [3.9]	-0.862	0.394
$STAI_{Trait}$	30.3 [10.0]	34.2 [9.3]	-1.230	0.227
Odor identification (Sniffin Sticks)	13.4 [1.0]	13.1 [0.8]	0.917	0.365
	Total $amount_{male}$	Total $amount_{female}$	t	p value
Relationship (committed relationship / single / no answer)	11 / 6 / 1	8 / 10 / 0	-0.589	0.560

SD = Standard deviation, df = 34

Supplementary Table S4. Description of the horror movie scenes.

Movie title	Scene content
The exorcist	A priest cleans cloths and then goes to a bed where a possessed child is lying. The child laughs and turns its head. The priest tries to exorcize the child's demons. The house begins to shake and the child's eyeballs twist until they turn white.
The shining	While the child repeats “redrum” and writes it on the door, the mother wakes up and notices that it says “Murder” the other way round. At the same time, a man tries to get into the bedroom with an axe. The woman and child try to escape through a window in the bathroom, but the mother is unable to get through the frozen window. She hangs on in the bathroom until her husband destroys the door with the axe. She wounds him with a knife.
Copycat	A female police officer walks down a corridor and searches for a murderer in the ladies' restroom. She opens a few doors in the process. She is ambushed by him and murdered with gunshots.
Child´s Play II (Chucky 2)	Chucky (a doll) is in a storeroom that a teacher unlocks. She calls for Andi and searches among the materials until Chucky scares her, stabs her with an air pump and makes her fall.
The blair witch project	Two people are looking for a third person in a house and garden. They film themselves in the process. In the course of the film, the two searchers get lost and all you can hear are shouts, from whom remains uncertain. At the end, the camera falls down and continues filming on its own.

Supplementary References

1 Alves Soares, T., Owsienko, D., Haertl, T. & Loos, H. M. Recovery rates of selected body odor substances in different textiles applying various work-up and storage conditions measured by gas chromatography-mass spectrometry. *Anal Chim Acta* **1252**, 341067, doi:10.1016/j.aca.2023.341067 (2023).

2 Kováts, E. Gas-chromatographische Charakterisierung organischer Verbindungen. Teil 1: Retentionsindices aliphatischer Halogenide, Alkohole, Aldehyde und Ketone. **41**, 1915-1932, doi:https://doi.org/10.1002/hlca.19580410703 (1958).
