# Supplementary material for: Enhanced sensitivity to odors due to chemosignals associated with anxiety
Source: Commun Chem. 2025 Apr 29;8:129. doi: 10.1038/s42004-025-01512-3 (PMC12041518; doi:10.1038/s42004-025-01512-3)
Supplement: Supplementary file 2 — Description of Additional Supplementary Files [file 42004_2025_1512_MOESM2_ESM.pdf]

Description of Additional Supplementary Files

File name: Supplementary Data 1

Description: Numerical source data for Figure 3 and S1. Participants are displayed in columns (Label 0 for Anxiety and 1 for Neutral) with features listed in rows.
