# Supplementary material for: Enhanced sensitivity to odors due to chemosignals associated with anxiety
Source: Commun Chem. 2025 Apr 29;8:129. doi: 10.1038/s42004-025-01512-3 (PMC12041518; doi:10.1038/s42004-025-01512-3)
Supplement: Supplementary file 4 — Reporting Summary [file 42004_2025_1512_MOESM4_ESM.pdf]

nature portfolio

Corresponding author(s): Annkatrin Wunder, Jessica Freiherr

Last updated by author(s): 24.03.2025

Reporting Summary

Nature Portfolio wishes to improve the reproducibility of the work that we publish. This form provides structure for consistency and transparency in reporting. For further information on Nature Portfolio policies, see our Editorial Policies and the Editorial Policy Checklist.

Statistics

For all statistical analyses, confirm that the following items are present in the figure legend, table legend, main text, or Methods section.

n/a	Confirmed	
☐	☒	The exact sample size (*n*) for each experimental group/condition, given as a discrete number and unit of measurement
☐	☒	A statement on whether measurements were taken from distinct samples or whether the same sample was measured repeatedly
☐	☒	The statistical test(s) used AND whether they are one- or two-sided<br>*Only common tests should be described solely by name; describe more complex techniques in the Methods section.*
☐	☒	A description of all covariates tested
☐	☒	A description of any assumptions or corrections, such as tests of normality and adjustment for multiple comparisons
☐	☒	A full description of the statistical parameters including central tendency (e.g. means) or other basic estimates (e.g. regression coefficient) AND variation (e.g. standard deviation) or associated estimates of uncertainty (e.g. confidence intervals)
☐	☒	For null hypothesis testing, the test statistic (e.g. *F*, *t*, *r*) with confidence intervals, effect sizes, degrees of freedom and *P* value noted<br>*Give P values as exact values whenever suitable.*
☒	☐	For Bayesian analysis, information on the choice of priors and Markov chain Monte Carlo settings
☒	☐	For hierarchical and complex designs, identification of the appropriate level for tests and full reporting of outcomes
☐	☒	Estimates of effect sizes (e.g. Cohen's *d*, Pearson's *r*), indicating how they were calculated

Our web collection on statistics for biologists contains articles on many of the points above.

Software and code

Policy information about availability of computer code

Data collection	No software was used
Data analysis	For data analysis of the GC-MS data, the freely accessible software MetaboAnalyst (version 6) was used. For analysing the behavioral data the freely accessible software JASP (JASP Team, Version 0.16.3.0) was used.

For manuscripts utilizing custom algorithms or software that are central to the research but not yet described in published literature, software must be made available to editors and reviewers. We strongly encourage code deposition in a community repository (e.g. GitHub). See the Nature Portfolio guidelines for submitting code & software for further information.

Data

Policy information about availability of data

All manuscripts must include a data availability statement. This statement should provide the following information, where applicable:

- Accession codes, unique identifiers, or web links for publicly available datasets
- A description of any restrictions on data availability
- For clinical datasets or third party data, please ensure that the statement adheres to our policy

The data of the behavioral study and the donor group is shared on the open science framework under the name of the corresponding author A.W.(DOI 10.17605/OSF.IO/EF2S8). Individual feature areas of the normalized GC-MS analysis can be viewed in the supplementary data sheet S1.Chromatographic data will be provided on request.

Research involving human participants, their data, or biological material

Policy information about studies with human participants or human data. See also policy information about sex, gender (identity/presentation), and sexual orientation and race, ethnicity and racism.

Reporting on sex and gender	In our manuscript we only use the term "sex", refering to the biological attribute as we obtained information abot the sex of the participants. Information on gender has not been obtained. The sex of the participant was accessed via self-reports. For the donation of body odors only participants with the sex female were included. For the behavioral study part an equally mixed group of females and males was tested. The results of the chemical analysis of the female sweat samples cannot be applied to both sexes, as studies indicate that the sweat composition of men and women is not the same. In order to prevent these sex-specific differences from interfering with the emotional differences, only female donors were tested. Sex-based analysis of the receiver group were not performed as sex was not part of the research question.
Reporting on race, ethnicity, or other socially relevant groupings	In our manuscript we do not report any information about race, ethnicity or other socially relevant groupings.
Population characteristics	See above.
Recruitment	Sweat donors were recruited via the Dental Clinic at the University of Erlangen-Nuremberg, surrounding dental practices, public notices, social media, an existing database and direct contact at the Erlangen Dental Clinic. Recruitment of receiving participants was carried out via notices in public buildings, posts on social media and an existing database of the working group.
Ethics oversight	Medical Faculty Ethics Review Board of the Friedrich-Alexander-Universität Erlangen-Nürnberg (no. 22-277-B)

Note that full information on the approval of the study protocol must also be provided in the manuscript.

Field-specific reporting

Please select the one below that is the best fit for your research. If you are not sure, read the appropriate sections before making your selection.

☐ Life sciences ☒ Behavioural & social sciences ☐ Ecological, evolutionary & environmental sciences

For a reference copy of the document with all sections, see nature.com/documents/nr-reporting-summary-flat.pdf

Behavioural & social sciences study design

All studies must disclose on these points even when the disclosure is negative.

Study description	This is an experimental study. It is a quantitative study.
Research sample	All participants were healthy, non-smokers and were neither pregnant nor breastfeeding. To obtain strong anxiety chemosignals, the chemosignals were obtained during a dental treatment of participants being afraid of the dentist. For the donation only female participants were included (see above)
Sampling strategy	The number of participants was based on the number in comparable studies. A G-Power analysis using results of previous research was also carried out.
Data collection	Exclusion criteria were checked via online questionaires. For the donation part of the study demografic data of the participants was accessed via printed out questionaires (pen and paper) and was digitalized later by the researchers. Questionnaires during the behavioral study were filled out online via a provided computer and results of the odor threshold test were handwritten by the researchers and digitalized afterwards. For the complex visual condition a horror movie was shown on a computer screen.Headphones were provided.<br>The researcher was not blinded to experimental condition nor study hypothesis.
Timing	Data was collected from November 2022 to November 2023. Chemical analysis was performed in the same time span.
Data exclusions	No data was excluded from the analysis.
Non-participation	No participants dropped out or declined participation.
Randomization	Participants were not allocated into experimental groups. However, the order of conditions and threshold test was randomized.

Reporting for specific materials, systems and methods

We require information from authors about some types of materials, experimental systems and methods used in many studies. Here, indicate whether each material, system or method listed is relevant to your study. If you are not sure if a list item applies to your research, read the appropriate section before selecting a response.

Materials & experimental systems

n/a	Involved in the study
☒	☐ Antibodies
☒	☐ Eukaryotic cell lines
☒	☐ Palaeontology and archaeology
☒	☐ Animals and other organisms
☒	☐ Clinical data
☒	☐ Dual use research of concern
☒	☐ Plants

Methods

n/a	Involved in the study
☒	☐ ChIP-seq
☒	☐ Flow cytometry
☒	☐ MRI-based neuroimaging

Plants

Seed stocks	not applicable
Novel plant genotypes	not applicable
Authentication	not applicable
